# Comparing In-Person to Videoconference-Based Cognitive Behavioral Therapy for Mood and Anxiety Disorders: Randomized Controlled Trial

**DOI:** 10.2196/jmir.2564

**Published:** 2013-11-19

**Authors:** Daniel R Stubbings, Clare S Rees, Lynne D Roberts, Robert T Kane

**Affiliations:** ^1^School of Psychology and Speech PathologyFaculty of Health SciencesCurtin University of TechnologyPerthAustralia

**Keywords:** telepsychology, videoconferencing, cognitive behavioral therapy, anxiety, mood disorder

## Abstract

**Background:**

Cognitive-behavioral therapy (CBT) has demonstrated efficacy and effectiveness for treating mood and anxiety disorders. Dissemination of CBT via videoconference may help improve access to treatment.

**Objective:**

The present study aimed to compare the effectiveness of CBT administered via videoconference to in-person therapy for a mixed diagnostic cohort.

**Methods:**

A total of 26 primarily Caucasian clients (mean age 30 years, SD 11) who had a primary Diagnostic and Statistical Manual of Mental Disorders, 4th edition text revision (DSM-IV-TR) diagnosis of a mood or anxiety disorder were randomly assigned to receive 12 sessions of CBT either in-person or via videoconference. Treatment involved individualized CBT formulations specific to the presenting diagnosis; all sessions were provided by the same therapist. Participants were recruited through a university clinic. Symptoms of depression, anxiety, stress, and quality of life were assessed using questionnaires before, after, and 6 weeks following treatment. Secondary outcomes at posttreatment included working alliance and client satisfaction.

**Results:**

Retention was similar across treatment conditions; there was one more client in the videoconferencing condition at posttreatment and at follow-up. Statistical analysis using multilevel mixed effects linear regression indicated a significant reduction in client symptoms across time for symptoms of depression (*P*<.001, *d*=1.41), anxiety (*P*<.001, *d*=1.14), stress (*P*<.001, *d*=1.81), and quality of life (*P*<.001, *d*=1.17). There were no significant differences between treatment conditions regarding symptoms of depression (*P*=.165, *d*=0.37), anxiety (*P*=.41, *d*=0.22), stress *(P*=.15, *d*=0.38), or quality of life (*P*=.62, *d*=0.13). There were no significant differences in client rating of the working alliance (*P*=.53, one-tailed, *d=*–0.26), therapist ratings of the working alliance (*P*=.60, one-tailed, *d*=0.23), or client ratings of satisfaction (*P*=.77, one-tailed, *d*=–0.12). Fisher’s Exact *P* was not significant regarding differences in reliable change from pre- to posttreatment or from pretreatment to follow-up for symptoms of depression (*P*=.41, *P*=.26), anxiety (*P*=.60, *P*=.99), or quality of life (*P*=.65, *P*=.99) but was significant for symptoms of stress in favor of the videoconferencing condition (*P*=.03, *P*=.035). Difference between conditions regarding clinically significant change was also not observed from pre- to posttreatment or from pretreatment to follow-up for symptoms of depression (*P*=.67, *P=*.30), anxiety (*P*=.99, *P*=.99), stress (*P*=.19, *P=*.13), or quality of life (*P*=.99, *P=*.62).

**Conclusions:**

The findings of this controlled trial indicate that CBT was effective in significantly reducing symptoms of depression, anxiety, and stress and increasing quality of life in both in-person and videoconferencing conditions, with no significant differences being observed between the two.

**Trial Registration:**

Australian New Zealand Clinical Trials Registry ID: ACTRN12609000819224; http://www.anzctr.org.au/ACTRN12609000819224.aspx (Archived by WebCite at http://www.webcitation.org/6Kz5iBMiV).

## Introduction

Approximately 20-25% of people are likely to suffer from a mood disorder during their lifetime [1]. They are a leading cause of disability for people aged 15-44, and the direct medical costs associated with unipolar depression have been estimated to be AUD $27,237 per person per year [2]. Anxiety disorders are also common, with approximately 29% of people likely to meet the diagnostic criteria during their lifespan [1]. Cognitive behavioral therapy (CBT) is an empirically validated psychological treatment that can be used to effectively treat mood and anxiety disorders. It is a time-limited and structured therapy that aims to help clients identify and reality-test unhelpful cognitions and correct maladaptive behavior [3,4]. Meta-analysis research has indicated that CBT is effective in the treatment of disorders such as depression [5], obsessive-compulsive disorder [6], generalized anxiety disorder [7], social phobia [8], panic disorder [9], and posttraumatic stress disorder [10]. Strong evidence also exists indicating that CBT can be effectively used to treat client groups presenting with a mixed array of mood and anxiety disorders [11].

At present, there is limited access to empirically validated treatments [12], particularly in remote locations. One way of increasing access is to provide services via remote media, such as telepsychology, which is the provision of psychological services via videoconference [13-15]. There is a substantial amount of evidence supporting the use of CBT to treat mood and anxiety disorders in-person; thus, it is important to determine if these findings extend to CBT administered via videoconference. Providing mental health services via videoconference for rural and remote populations has been reported to be cost effective [16-18], have high satisfaction [19,20], be used to reduce referrals to in-patient clinics [21], and offer diagnosis results comparable outcomes to in-person [22,23]. Interaction via videoconference has also been shown to reduce depression and loneliness in elderly nursing home residents [24].

Some evidence exists indicating that manualized CBT provided via telepsychology for restrictive diagnostic profiles can be effective [25-28]. Bouchard et al [25] conducted a controlled trial comparing a manualized CBT treatment for panic and agoraphobia via videoconference (n=11) to in-person (n*=*10). The results indicated that 81% (9/11) of participants in the videoconferencing condition were panic-free at posttreatment and 91% (10/11) at 6-month follow-up. The differences between conditions with regards to client symptoms and quality of the working alliance were not significant. Mitchell et al [27] conducted a randomized controlled trial of CBT for the treatment of bulimia nervosa and compared the clinical outcomes obtained in-person to via videoconference. Six doctoral-level students administered therapy, and 128 participants were involved over a 4-year time period. Again, no significant differences were observed between the in-person and videoconferencing condition with regards to reduction in symptomology, therapeutic alliance, and treatment retention. Morland et al [28] conducted a study involving 125 participants with posttraumatic stress disorder randomly assigned to either in-person or videoconference-based group therapy for anger management. Similarly, the findings indicated that the clinical outcomes obtained via videoconference were not inferior to what were obtained in-person.

Some studies have been conducted that involve a mixed diagnostic client cohort. Day and Schneider [29] conducted a randomized controlled trial comparing the effectiveness of CBT when administered either in-person, via 2-way audio or via videoconference for a cohort of clients with a variety of presenting issues. All treatment sessions were conducted at the same location. The study involved 16 clinicians and 80 clients, and the most frequent presenting concerns pertained to body image, family relationships, self-esteem, and work/school issues. The intervention was effective in all conditions, and the results did not indicate any significant difference between treatment modalities. Despite these encouraging findings, the intervention included only five treatment sessions, psychiatric conditions were not diagnosed, reliable change and clinical significance were not analyzed, and a multivariate analysis was used to address a series of univariate research questions. Griffiths, Blignault, and Yellowlees [30] reported the details of a study involving 15 clients presenting with depression and/or anxiety who were treated with 6-8 sessions of CBT by their case managers. The pre-post treatment data indicated that the clients’ mental health was significantly better after treatment, but there was no in-person control condition. In a more recent study involving an in-person randomized comparison condition, Dustan and Tooth [31] investigated clinical outcomes for 6 clients (3 in each condition) that presented with either an anxiety or mixed anxiety-depression disorder. Two trainee psychologists provided 6-8 sessions of CBT, and symptoms were measured pre-, post, and 1-month follow-up. All 3 clients in the videoconferencing condition showed significantly reduced symptomology at follow-up, but due to the small sample size, an analysis comparing in-person to videoconference-based outcomes could not be made. Given these limitations, further videoconference-based research is needed to extend in-person research pertaining to mood and anxiety disorders [11].

Research has indicated that when CBT is successful in reducing the symptoms of a psychological disorder, clients typically experience a concurrent increase in quality of life [32]. Changes in quality of life as a result of treatment administered via digital media have been addressed in some contexts [33], but research specifically pertaining to videoconferencing and quality of life is limited [34]. Hence further research is recommended to address this gap in the literature.

The present study aimed to investigate whether CBT administered via videoconference produces comparable clinical outcomes to treatment provided in-person. The treatment will be focused on addressing anxiety and mood disorders including participants that present with comorbid diagnosis. This study used a randomized, active control design, structured clinical assessments, with symptoms measures administered at pretreatment, posttreatment, and at 6 weeks following treatment.

##  Methods

### Participants

**Figure 1:**
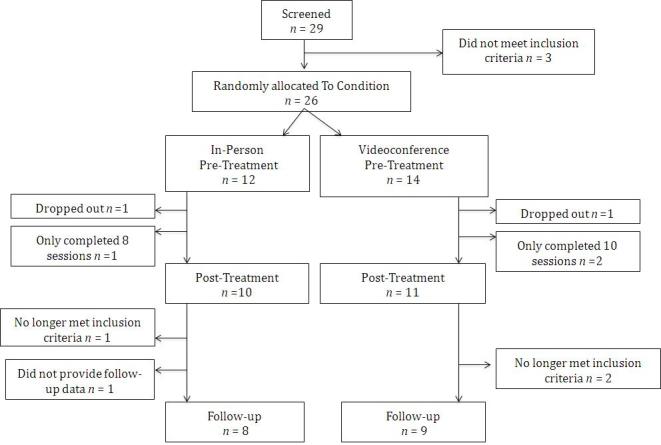
The number of participants and their flow throughout the study.

The Curtin University ethics committee approved this study and all participants provided signed consent. Participants were recruited via the Curtin University Psychology Clinic and were either self-referred or referred from community health agencies via telephone, letter, fax, or email. Participant recruitment began in January 2010 and ended in April 2011. The recruitment ended because no more time was available for this activity during the course of the degree. We recruited 29 participants but 3 did not meet the inclusion criteria. Inclusion criteria consisted of a primary diagnosis of a Diagnostic and Statistical Manual of Mental Disorders, 4th edition text revision (DSM-IV-TR) [35] Axis-I disorder, aged 18-65 years old, and living in Perth, Western Australia. Exclusion criteria included a DSM-IV-TR [35] diagnosis of anorexia, psychosis (past or present), or a personality disorder as the primary diagnosis, as well as any self-harm or suicidal behaviors currently receiving psychotherapy and/or involvement in legal proceedings. Figure 1 displays the participant flow over the course of the study. All assessments, treatment, and data collection were conducted at the university clinic.

The sample included 26 participants, aged 18-59 years (mean 30 years, SD 11 years). The videoconferencing condition included 6 males and 8 females; the in-person condition included 5 males and 7 females. An in-person clinical interview along with the Structured Clinical Interview for the DSM (SCID) [36,37] was used to screen the participants. The Interference of Severity Scale adapted from the Anxiety Disorders Interview Schedule [38] was used to determine which disorder should be the primary focus of treatment. The average number of presenting disorders in both conditions was 3, and there were only 2 clients who presented with only 1 diagnosis. The number of clients in each condition who met the criteria for various Axis-I and Axis-II disorders is displayed in Tables 1 and 2. No data were collected regarding clients’ prior experience with computers and videoconferencing technology. Six participants reported that they were on antidepressants before starting the study. A record of medication usage by participants was not taken, but they were asked not to change their medication dosage during the course of the study.

**Table 1:** The number of clients that met the criteria for Axis-I disorders at pretreatment.

		Primary Axis-I disorder			Comorbid Axis-I disorder	
		IP^a^	VC^b^		IP	VC
**Axis-I mood disorders**						
	Major depressive disorder	5		1	3	4
	Dysthymic disorder			1	3	3
	Cyclothmic disorder					1
**Axis-I anxiety disorder**						
	Panic with agoraphobia			1		
	Panic without agoraphobia	1			2	
	Social phobia			1	1	
	Obsessive-compulsive disorder	4		8	1	1
	Posttraumatic stress				2	
	Generalized anxiety	2			3	2
	Hypocondriasis			1	1	1
**Other Axis-I disorders**						
	Adjustment disorder			1		
	Eating disorder NOS (binge eating)				2	
	Alcohol dependence					1
	Cannabis dependence					1
	Dysparenuia				1	
	Impulse control disorder (not otherwise specified)					1
	Attention-deficit hyperactivity				2	
	Amphetamine-induced psychotic disorder					1
	Substance-induced mood disorder with manic features					1

^a^IP=In-person.

^b^VC=Videoconference.

**Table 2:** The number of clients that met the criteria for Axis-II disorders at pretreatment.

Axis-II disorders	In-person	Videoconference
Schizotypal personality disorder		1
Borderline personality disorder	1	
Narcissistic personality disorder	1	1
Avoidant personality disorder	6	4
Obsessive-compulsive	5	9

### Treatment Protocols

For each client, an individualized CBT formulation was devised. Manualized CBT interventions were used as a guide to planning and implementing treatment for specific disorders such as panic/agoraphobia [39], depression/dysthymia [3], hypochondriasis [40], generalized anxiety [41], and obsessive-compulsive disorder [42]. Other conditions were treated with standard CBT techniques such as psychoeducation, symptom monitoring, cognitive restructuring, and exposure exercises relevant to their presenting symptoms. The interventions were focused on addressing symptoms of the primary Axis-I diagnosis. Axis-II conditions were noted in the assessment sessions but were not targeted during the course of treatment.

### Self-Report Measures

The Depression Anxiety and Stress Scale (DASS) [43] was used to measure global clinical symptoms. The Quality of Life Enjoyment and Satisfaction scale (QLES) [44] was used to measure changes in quality of life as a result of treatment. Given that a mixed diagnostic cohort was used, disorder specific measures for the primary presenting condition were also administered. These included the Beck Depression Inventory-II [45], Obsessive-Compulsive Inventory [46], Health Anxiety Questionnaire [47], Penn State Worry Questionnaire [48], and the Anxiety Sensitivity Index [49]. The DASS, QLES, and disorder-specific measures were administered before treatment, after treatment, and 6 weeks after treatment had ended. The Working Alliance Inventory Short Form [50] was used to measure the strength of the working alliance from both the clients’ and therapists’ perspectives. Client satisfaction was measured using the shortened Client Satisfaction Questionnaire [51]. The Telehealth Satisfaction Questionnaire [52] was used to measure client satisfaction specifically with the technology. Both the Working Alliance Inventory Short Form and the Client Satisfaction Questionnaire were administered at posttreatment to all clients; the Telehealth Satisfaction Questionnaire was administered at posttreatment only to clients allocated to the videoconferencing condition. All questionnaires were completed by hand.

###  Apparatus

Three Mac computers were used during the course of this study. All three computers were connected via ethernet (100 Mbps/sec) within the same building. The program used to conduct the videoconferencing was iChat, versions 4.0.9 to 5.0.3. When the study was conducted, the interactions via iChat were encrypted, but this feature was removed in Mac OS X 10.7. Therefore iChat may no longer be suitable for future telepsychology research.

###  Procedure

The initial assessment consisted of a clinical interview, the SCID, and administration of the DASS and QLES. The therapist (primary author) who conducted the screening, diagnosis, and treatment was a provisionally registered (trainee) clinical psychologist doctoral student. Following the initial assessment, clients were randomly allocated to receive treatment either in-person or via videoconference. Simple random allocation was used to determine assignment to condition. This was achieved by generating a randomized list of binary numbers [53]. The generated list was calculated on the basis of 200 potential participants, two groups, and one repeat of randomization. Clients in the in-person condition began “treatment as usual”. Clients in the videoconferencing condition were instructed to walk into the treatment room at the clinic during their allotted time and sit in front of the computer. After the initial diagnosis session, clients allocated to the videoconferencing condition had no other in-person contact with the treating therapist. Materials, such as new homework diaries, were placed in the treatment room before the client arrived. Participants in the videoconferencing condition shared the content of their homework diaries orally; therefore, they did not need to hand in material to the therapist. All of the client sessions were recorded so that the fidelity and credibility of the therapists’ diagnosis and treatments could be monitored. The clinical research supervisor (the second author, a registered clinical psychologist) provided weekly supervision for a minimum of 1 hour to the practicing psychologist (primary author) throughout the duration of data collection. During supervision, videotaped sessions of all clients in the study were observed and checked for adherence to CBT protocols and that the majority of the session time adhered to the protocol. Clients who took part in the study were offered 12 weekly 1-hour sessions and an additional follow-up session 6 weeks after the 12th session. A fee for clinical services was not charged, and no reimbursement for their time was provided. In-session exposure activities were limited to tasks that could be accomplished within the treatment room. Sessions times were arranged directly with the therapist; therefore, there was no need for participants in either condition to interact with support staff such as a receptionist. Participants were aware that the therapist was located within the same building. The CONSORT-EHEALTH guidelines for improving and standardizing the reporting of Web-based and mobile health interventions were used as a guide throughout this research [54].

### Research Design and Data Analysis

The research design included one nominal fixed effect (condition: in-person versus videoconference), one ordinal fixed effect (time: pre, post, follow-up), one nominal random effect (participant), and four scale outcomes (depression, anxiety, stress, and quality of life). The data generated by this design were analyzed with a multilevel mixed effects linear regression model [55] as implemented through the Generalized Linear Mixed Models procedure in the SPSS software, version 19 [56]. The analysis was “multilevel” in the sense that it was conducted within the context of a hierarchical data structure in which time was nested within participant. In order to optimize the likelihood of convergence, a separate analysis was run for each of the four outcomes. With a predicted large effect size of *d*=0.5, an alpha level of .05, desired power of 0.8, and a correlation of 0.6 between repeated measures, the estimated total sample size using G-Power [57] was 26 (13 participants per condition). The original intent was to have sufficient power to detect a small difference, which would have required 115 participants. However, this target became impractical given the time and resource constraints.

##  Results

###  Attrition

Of the clients eligible to take part in the study, 81% (21/26) completed the full course of treatment, and 65% (18/26) completed the follow-up data. Figure 1 shows the participant flow over the course of the study. In the videoconferencing condition, 2 of the participants completed only 10 sessions: the first, because they moved overseas, and the second stated that they had done all the change they felt capable of doing at that time. In the videoconferencing condition, 2 of the participants were no longer eligible for inclusion in the study at follow-up: the first, because they required ongoing treatment after the posttreatment data had been collected and the second decided to begin medication in the final week of treatment. In the in-person condition, 1 participant dropped out after nine sessions and no reason was given. Also in the in-person condition, 1 participant was no longer eligible to be included in the study at follow-up because they commenced additional ongoing psychotherapy treatment after the initial 12-weeks of treatment in the study. The analyses were conducted twice: once with only the participants that completed all 12 sessions and once with all the participants that completed eight or more sessions. The results of the two analyses provided the same findings; therefore, participants who completed eight or more sessions were included in the final analyses.

###  Comparison of Participant Characteristics at Pretreatment

There was a small nonsignificant difference between the mean ages of participants in the in-person condition (mean 29.67, SD 9.31) and the videoconferencing condition (mean 31.93, SD 13.33) (*t*_24_=–0.49, *P*=.63, two-tailed, 95% CI -11.73 to 7.21, *d*=–0.19). A Pearson chi-square test of contingencies indicated that gender was equally distributed across conditions χ^2^_1_=0.004 (N=26), *P*=.95, and the association between gender and condition was very small with phi=.01. The difference between conditions at pretest was not significant on the DASS depression subscale (*t*_23_=0.05, *P*=.96, two-tailed, 95% CI -8.28 to 8.56, *d*=0.02), the anxiety subscale (*t*_23_=0.37, *P*=.71, two-tailed, 95% CI -5.98 to 8.57, *d*=0.15), the stress subscale (*t*_23_=0.19, *P*=.85, two-tailed, 95% CI -8.36 to 6.98, *d*=–0.08), or on the QLES (*t*_24_=0.28, *P*=.78, two-tailed, 95% CI -0.47 to 0.62, *d*=0.10).

### Primary Analyses

**Figure 2:**
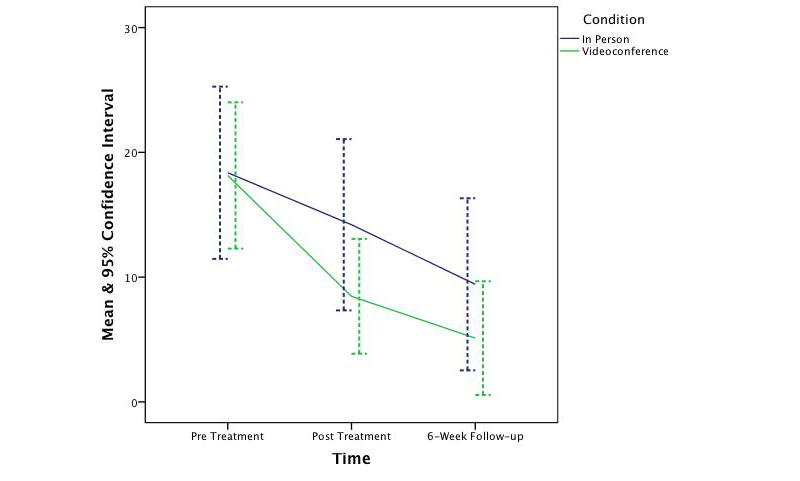
The change in symptoms of depression across time and condition.

**Figure 3:**
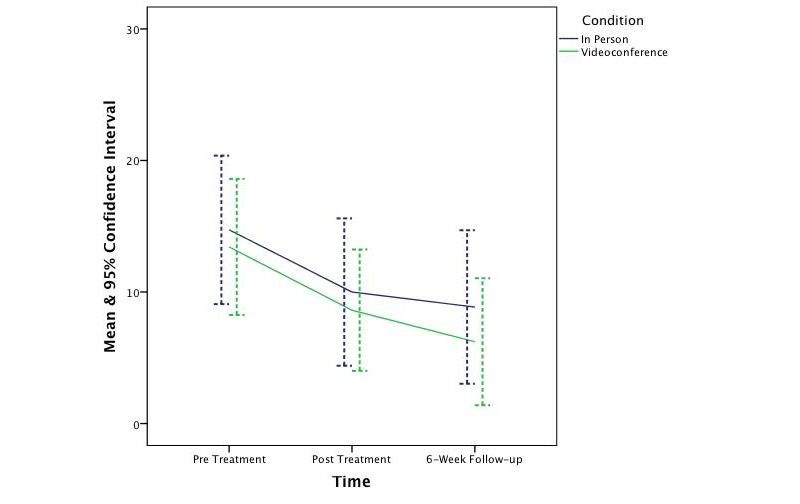
The change in symptoms of anxiety across time and condition.

**Figure 4:**
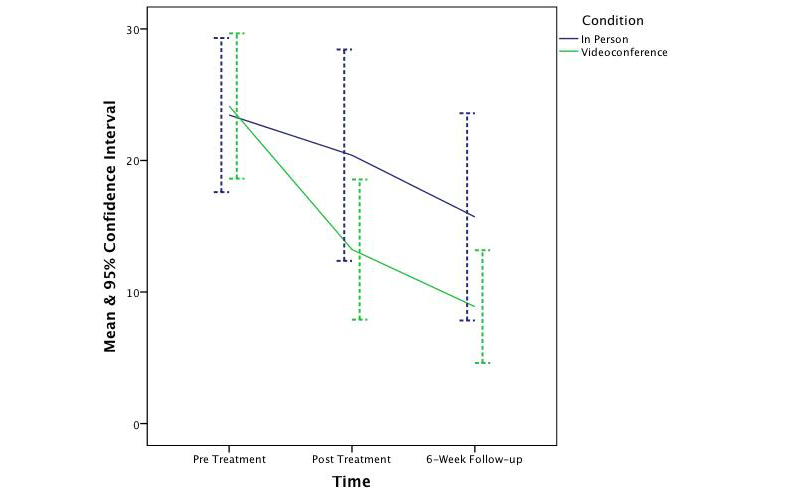
The change in symptoms of stress across time and condition.

**Figure 5:**
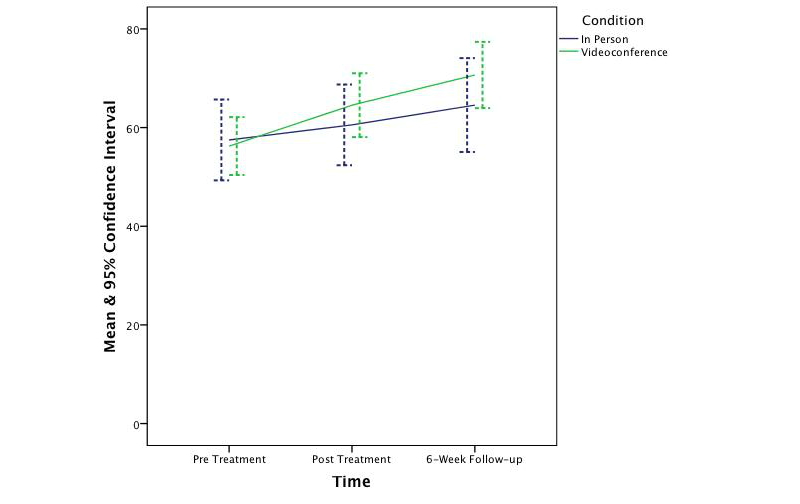
The change in quality of life across time and condition.

In order to reduce the chances of a Type-1 error, the Bonferroni correction was applied throughout the four analyses making the alpha level .0125. The descriptive statistics for the in-person and videoconferencing conditions are reported in Table 3. The interaction between time and condition was not significant for all three DASS subscales (Depression *F*_2,58_=1.77, *P*=.18; Anxiety *F*_2,58_=0.36, *P*=.7; and Stress *F*_2,58_=4.19, *P*=.02), and the QLES (*F*_2,62_=0.82, *P*=.45); therefore, the main effects can be interpreted without qualification. There was a significant main effect for time on all three DASS subscales (Depression *F*_2,58_=14.47, *P*<.001; Anxiety *F*_2,58_=9.34, *P*<.001; and Stress *F*_2,58_=23.70, *P*<.001) and the QLES (*F*_2,62_=10.64, *P*<.001). The effect sizes were large throughout (*d*=1.41, 1.14, 1.81, and 1.17 respectively). In contrast, there was no significant main effect for condition on any of the DASS subscales (Depression *F*_1,58_=1.98, *P*=.16; Anxiety *F*_1,58_=0.69, *P*=.41 and Stress *F*_1,58_=2.11, *P*=.15) or the QLES (*F*_1,62_=0.25, *P*=.62). The effect sizes ranged from small to medium (*d*=0.37, 0.22, 0.38, and 0.13 respectively). Figures 2-5 depict the upper and lower bound of the 95% confidence intervals around the mean scores at pre, post, and 6 weeks following treatment in both conditions for the three DASS subscales and the QLES.

**Table 3:** Descriptive statistics for the scale measures in the in-person and videoconferencing conditions.

Scale measure		In-person condition					Videoconferencing condition				
		n	Mean	SD	Min	Max	n	Mean	SD	Min	Max
**DASS depression subscale**											
	Pre	11^a^	18.36	10.27	4	32	14	18.14	10.15	6	42
	Post	10	14.2	9.59	6	34	13	8.46	7.62	0	28
	Follow-up	7	9.43	7.46	2	22	9	5.11	5.93	0	18
**DASS anxiety subscale**											
	Pre	11^a^	14.73	8.4	6	32	14	13.43	8.96	0	26
	Post	10	9.09	8.02	0	26	13	8.62	7.63	2	26
	Follow-up	7	7.75	6.63	0	18	9	6.22	6.28	0	16
**DASS stress subscale**											
	Pre	11^a^	23.45	8.72	10	36	14	24.14	9.56	8	38
	Post	10	18.55	12.3	0	42	13	13.23	8.81	2	30
	Follow-up	7	13.75	9.65	0	34	9	8.89	5.58	0	18
**QLES**											
	Pre	12	3.38	0.76	2.06	4.88	14	3.31	0.59	2.29	4.29
	Post	11	3.58	0.72	2.24	4.71	13	3.81	0.62	2.76	4.94
	Follow-up	8	3.85	0.76	2.65	5.88	9	4.15	0.51	3.41	4.88
Credibility of therapy		11	34.14	3.73	25	38	13	34.69	4.52	23	40
Working alliance: Client		11	6.14	0.45	5.5	7	12^b^	6.33	0.89	3.75	7
Working alliance: Therapist		11	5.89	0.41	4.92	6.417	13	5.74	0.83	3.58	6.75
Client satisfaction		11	93.21	6.37	78.13	100	13	94.23	10.04	65	100

^a^The DASS data for one of the participants in the in-person condition was removed because it was invalid at pretreatment.

^b^The Working Alliance-Client data for one of the participants in the videoconferencing condition was removed because it was invalid.

### Secondary Analyses

The secondary analyses were administered only at posttest. There were no significant differences between conditions in client ratings of the Working Alliance Inventory Short Form (*t*_21_=–0.63, *P*=.53, one-tailed, *d=–*0.26), or in therapist ratings (*t*_22_=0.53, *P*=.60, one-tailed, *d*=0.23), and client ratings of the Client Satisfaction Questionnaire (*t*_22_=–0.29, *P*=.77, one-tailed, *d*=–0.12). Clients in the videoconference-based condition who completed 10 or more sessions were included in the Telehealth Satisfaction Questionnaire analysis (n=13). Scores ranged from 61.4 to 100 (maximum score is 100), with a mean of 90.44 (SD 11.11).

### Reliable Change and Clinical Significance

Reliable change and clinical significance calculations [58] were conducted on the DASS subscales, the QLES, and disorder specific measures. Normative data were used to calculate the cutoff criteria [3,43-47,59-64]. Tables 4-6 display the proportion of clients in each condition that met the respective criteria for the three DASS subscales and the QLES respectively.

Fisher’s Exact test [65] was used to compare the proportion of participants meeting the criteria for reliable change and clinical significance in each condition. With regards to reliable change, Fisher’s Exact was not significant from pre- to posttreatment or from pretreatment to follow-up for the depression (*P*=.41, *P*=.26) and anxiety (*P*=.60, *P*=.99) DASS subscales, and the QLES (*P*=.65, *P*=.99). Fisher’s Exact *P*, however, was significant for the stress DASS subscale from both pre- to posttreatment (*P*=.03) and from pretreatment to follow-up (*P*=.03) in favor of the videoconferencing condition. With regards to clinically significant change, the difference between conditions was not significant from pre- to posttreatment or from pretreatment to follow-up for the depression (*P*=.67, *P=*.30), anxiety (*P*=.99, *P*=.99), and stress (*P*=.19, *P=*.13) DASS subscales or the QLES (*P*=.99, *P=*.62). Table 7 displays the number of clients that upon discharge met the criteria for both reliable change (improved) and clinically significant change (recovered) for the various disorder specific measures administered in each condition. However, not all clients presented with severe symptoms at pretreatment. The proportion of clients whose symptoms were identified in the moderate to severe range at pretreatment are displayed in Table 8.

**Table 4:** Rates of clinical improvement from pre- to posttreatment for the DASS.

Clinical status	Pretreatment to posttreatment					
	Depression		Anxiety		Stress	
	IP^a^	VC^b^	IP	VC	IP	VC
Recovered	3/10	5/13	1/10	2/13	2/10	7/13
Improved	4/10	8/13	1/10	3/13	2/10	9/13
Unchanged	6/10	4/13	9/10	10/13	8/10	4/13
Deteriorated	None	1/13	None	None	None	None

^a^IP=In-person.

^b^VC=Videoconference.

**Table 5:** Rates of clinical improvement from pretreatment to follow-up for the DASS.

	Pretreatment to follow-up					
Clinical status	Depression		Anxiety		Stress	
	IP^a^	VC^b^	IP	VC	IP	VC
Recovered	3/7	7/9	2/7	2/9	2/7	7/9
Improved	4/7	8/9	2/7	2/9	2/7	8/9
Unchanged	4/7	1/9	5/7	7/9	5/7	1/9
Deteriorated	None	None	None	None	None	None

^a^IP=In-person.

^b^VC=Videoconference.

**Table 6:** Rates of clinical improvement from pre to posttreatment and follow-up for the QLES.

Clinical status	Pre to post		Pre to follow-up	
	IP^a^	VC^b^	IP	VC
Recovered	2/11	2/13	3/8	3/9
Improved	2/11	4/13	3/8	4/9
Unchanged	9/11	9/13	4/8	5/9
Deteriorated	None	None	1/8	None

^a^IP=In-person.

^b^VC=Videoconference.

**Table 7:** The number of clients in each condition identified as recovered on the disorder specific measures.

Condition	Primary diagnosis	Disorder specific measure	Change status	n
**Videoconference**				
	Obsessive- compulsive disorder	Obsessive-Compulsive Inventory	Recovered	4/9
			No change^a^	2/9
			No change	3/9
	Depression	Beck Depression Inventory-II	Recovered	1/2
			No change^a^	1/2
	Hypochondriasis	Health Anxiety Questionnaire	Recovered	1/1
	Generalized anxiety disorder	Penn State Worry Questionnaire	Recovered	1/1
**In-person**				
	Depression	Beck Depression Inventory-II	Recovered	2/4
			No change^a^	1/1
			No change	1/1
	Obsessive- compulsive disorder	Obsessive-Compulsive Inventory	Recovered	1/3
			No change^a^	1/3
			No change	1/3
	Generalized anxiety disorder	Penn State Worry Questionnaire	Recovered	2/3
			No change	1/3
	Panic disorder	Anxiety Sensitivity Index	Recovered	1/1

^a^Although some clients were not identified as recovered or improved on their disorder specific measure, they were identified as recovered on the DASS.

**Table 8:** The severity of client symptoms at pretreatment.

Symptom	In-person (n=12) n (%)		Videoconference (n=14) n (%)	
	Moderate-severe	Normal-mild	Moderate-severe	Normal-mild
Depression	7 (58)	5 (42)	7 (50)	7 (50)
Anxiety	8 (76)	4 (33)	9 (64)	5 (36)
Stress	10 (83)	2 (17)	11 (79)	3 (21)
Quality of life	10 (83)	2 (17)	11 (79)	3 (21)

## Discussion

### Principal Findings

The findings of this controlled trial indicate that CBT was effective in significantly reducing symptoms of depression, anxiety, and stress and increasing quality of life in both in-person and videoconferencing conditions. Furthermore, outcomes for the videoconferencing group were comparable to the in-person group, with no significant differences being observed between the two conditions. This finding is consistent with prior CBT meta-analysis research [66,67] and prior telepsychology research [18,25-27,68-71]. Although the sample was not large enough to permit a test of noninferiority, the 95% confidence intervals around the mean scores across time and condition suggest CBT treatment administered via videoconference is not inferior to treatment provided in-person. This finding is congruent with prior noninferiority analyses [28,70]. It is also important to note that the clinical outcomes at posttreatment and follow-up were better, albeit not significantly better, in the videoconferencing condition than in the in-person condition. Therefore, if this study had a greater power to detect an effect, it is unlikely this study would have shown videoconference-based CBT to be inferior to in-person.

Client and therapist ratings of the working alliance were high in both the in-person and videoconferencing conditions. The difference between conditions was not significant, and the effect size was trivial. These findings suggest that CBT treatment via videoconference does not compromise the working alliance, which is congruent with prior telepsychology research [26,72]. The results also indicated that client satisfaction was high and did not significantly differ between conditions, which again is congruent with prior research [19,20,73].

### Strengths and Limitations

The inclusion of a mixed diagnostic client cohort with comorbid diagnosis (as measured in a structured clinical interview) is a strength of this study. A client group with a range of disorders and comorbidities helps in generalizing the findings to real-world clinical practice populations and thus provides empirical support for the effectiveness of CBT-oriented telepsychology. Second, the same therapist treated all clients, and thus this variable was held constant. By including only 1 therapist in the study, the difference between conditions cannot be attributed to differences between therapists. A further strength of this study is that it was conducted on a high-speed local network. As a result of conducting the study on a system that is presently faster than the average Australian Internet speed, it may be a longer period of time before the conclusions of this study become outdated. Also, all of the participants in both conditions received treatment in the same building; consequently, this study was able to demonstrate that satisfaction remained high even when travel was not a contributing factor in the ratings of service satisfaction. No technological problems occurred during the study, and clients did not report any complaints about the technology.

Although this study has successfully extended previous telepsychology research [25-27,30,69,70], there are some limitations. First, because only 1 therapist was included in this study, it remains unclear as to whether or not the findings generalize to other therapists. Second, the diagnosis of clients in both conditions was conducted in-person by the same therapist who conducted the subsequent treatment. It is possible that rapport and a therapeutic alliance began in this initial in-person diagnosis session and influenced clinical outcomes. Third, the sample size was not large enough to conduct an analysis of noninferiority. The limited small sample size also weakens the generalizability of the findings. Fourth, there was a high risk of bias in the study given that the principal investigator was also responsible for treatment and assessing participants. However, clinical supervision was provided regarding both diagnosis and treatment throughout the study, and sessions were recorded and watched by the supervising clinician to temper this potential bias. Fifth, the diagnosis of participants was not evenly distributed across conditions, and as a result, the two groups might not have been equally matched. Sixth, as is the case in most research studies, the participants did not have to pay for the services, and this may have led them to provide socially desirable answers on the questionnaires. A replication study involving a fee for service would be able to clarify if this is an issue. Seventh, the average age of participants was 30; therefore, it is likely that most participants were already familiar with computer technology, which may have influenced their acceptance of the media.

Despite being a strength of the study, the inclusion of a mixed diagnostic cohort combined with a small sample size means there was a large degree of variability across the clients, which may have reduced the power of the study. Also, Bonferroni corrections were applied to reduce the risk of a Type-I error, further reducing the power of the study to detect significant interactions between time and condition. While providing promising results, a larger study is still needed to more thoroughly address the primary research question.

Not all outcomes in this study were favorable. Only 10% (1/10) of clients in the in-person condition and 33% (2/13) of participants in the videoconference condition were identified as meeting the criteria for a clinically significant reduction in anxiety. These results could be because 33% (4/12) and 36% (5/14) (in-person and videoconference respectively) of participants were identified on the DASS as having normal-to-mild levels of anxiety before beginning treatment. Alternatively, the therapist may not have been as competent at treating symptoms of anxiety compared to depression for which 43% (3/7) and 77% (7/9) of participants (in-person and videoconference respectively) were identified as meeting the criteria for clinically significant change. The presence of comorbid personality disorders may also have hampered treatment outcomes. In either case, a larger trial involving multiple therapists, systematic monitoring of treatment protocol adherence and a single diagnosis may help to overcome these issues in future research.

Some psychotherapy procedures via videoconference required creative modification. In-session exposure exercises via videoconference had to be limited to what could be conducted within the clinic room. For example a client working through exposure-response prevention activities pertaining to germs had to find items they found troubling that were in their treatment room as opposed to in-person sessions that could have the freedom to move outside of the treatment room. Another change was that cognitive restructuring worksheets had to be conducted on a digital Microsoft Word document on the clients’ computer but controlled from the therapist’s computer. These examples show that treatment via videoconference may alter how some interactions are conducted but does not prevent essential treatment components from being provided.

### Conclusions

It is important to consider the findings of this study within the context of the social justice, financial, and practical issues facing remote psychological services in Australia. In Australia, there is a disparity in access to psychological services between people who live in cities and those who live in rural and remote areas [74]. Rural and remote populations have higher rates of accidents, suicide, and exposure to violence [75] as well as increased rates of risk factors known to play a role in the emergence of psychological dysfunction, including poor physical health, obesity, smoking, drug abuse, high blood pressure, and poor nutrition [76]. As for the indigenous populations in rural and remote communities, the state of affairs is considerably worse where the rates of mortality and morbidity are approximately 2-4 times higher than for nonindigenous Australians [77]. People in rural and remote Australia are in the highest need of specialized mental health services; however, due to the lack of specialized clinicians working in these areas, the accessibility of those services frequently range from little to none [76]. Hence, there is a substantial demand for videoconference-based psychological services. However, there is often insufficient technological infrastructure to provide reliable high-speed broadband Internet communications, particularly in remote areas where there can be less than one person per square kilometer [78]. It is hoped that with the rollout of the National Broadband Network in Australia more rural and remote areas will gain access to high-quality videoconferencing technology. Prior research [17,79] has indicated that providing remote services via videoconference can be cost effective under some circumstances, but further research is needed to determine if it is also cost effective for small isolated indigenous communities. Further research is needed to identify the logistical challenges of providing videoconference-based CBT to rural and remote communities and to determine which method of service delivery is best suited to the needs of specific communities. Telepsychiatry services through medicare in Australia have increased from 15 consultations in 2002/3 to 2555 in 2010/11 [80]. This indicates that when available, both service providers and clients are willing to use the technology. Hopefully, this trend will extend to telepsychology services in the future as access to the technology increases.

In conclusion, this study provides support for the effectiveness of CBT via telepsychology for an adult client cohort and preliminary support of noninferiority. The results of this controlled trial provide important evidence to justify the greater use of videoconferencing to bridge the gap in service provision to populations who would otherwise not receive effective psychological treatments. Future research should continue to involve mixed diagnostic client cohorts with comorbid diagnoses and be directed at conducting large multisite randomized controlled trials that employ noninferiority design and analysis procedures.

## Abbreviations

CBT: cognitive-behavioral therapy
DASS: Depression Anxiety and Stress Scale
DSM-IV-TR: Diagnostic and Statistical Manual of Mental Disorders, 4th edition text revision
QLES: Quality of Life and Satisfaction Questionnaire
